# Supplementary material for: Functional redundancy compensates for decline of dominant ant species
Source: Nat Ecol Evol. 2025 Apr 22;9(5):779–88. doi: 10.1038/s41559-025-02690-y (PMC12066353; doi:10.1038/s41559-025-02690-y)
Supplement: Supplementary file 1 — Supplementary Tables 1–5 and Notes 1–6. [file 41559_2025_2690_MOESM1_ESM.pdf]

nature ecology & evolution

Supplementary information https://doi.org/10.1038/s41559-025-02690-y

Functional redundancy compensates for decline of dominant ant species

In the format provided by the authors and unedited

Supplementary information

Supplementary Table 1. Model comparison of five candidate Generalized Additive Mixed Models (GAMMs) of increasing complexity, testing the shape of the relationship between species richness and functional richness in control versus ant suppression treatments (Figure 1). We tested (1) the null intercept-only model, (2) a treatment-only model, (3) a species richness(SR)-only model, (4) a model with different treatment-level intercepts but the same SR relationships, and (5) a model with different treatment-level intercepts and different SR relationships for control versus suppression treatments. All models incorporated the same random effects of plot nested within spatial block to account for repeated measurements per plot within the split plot design. Models were compared using Akaike Information Criterion (AIC values). Loglik = model log likelihood; k = number of model parameters; ΔAIC = difference in AIC value from the model with lowest AIC value; AIC weight = probability that the model is the best-fit model; R-squared = model coefficient of determination.

Model	Name	k	LogLik	AIC	ΔAIC	AIC weight	R-squared
5	treatment * SR(smooth)	9	224.21	-428.14	0.00	0.87	0.66
4	treatment + SR(smooth)	7	219.89	-424.39	3.75	0.13	0.63
3	SR(smooth) only	6	212.21	-411.40	16.74	0.00	0.51
2	treatment only	5	181.02	-351.32	76.83	0.00	0.05
1	intercept only (null)	4	178.53	-348.58	79.56	0.00	0.00

Supplementary Table 2. Total abundances of non-target ant species sampled in pitfall traps in the year following suppression of the three dominant target species (Nov 2014 – Nov 2015). Data support the analysis of non-target species responses to dominant species removal shown in Extended Data Figure 4, so the target species *Iridomyrmex purpureus*, *Pheidole ampla perthensis*, and *Tetramorium impressum* are not included here.

**Subfamily**	**Species**	**Species code**	**Abundance in control plots**	**Abundance in suppression plots**
Formicinae	*Camponotus lownei* Forel, 1895	cam.low	0	4
Formicinae	*Camponotus nigriceps* Smith, F., 1858	cam.nig	14	0
Formicinae	*Camponotus terebrans* Lowne, 1865	cam.ter	1	3
Myrmicinae	*Cardiocondyla atalanta* Forel, 1915	car.atl	8	12
Dorylinae	*Lioponera incontentus* Brown, 1975	cer.inc	11	0
Dolichoderinae	*Iridomyrmex bicknelli* Emery, 1898	iri.bic	191	242
Dolichoderinae	*Iridomyrmex brunneus* Forel, 1902	iri.bru	45	60
Dolichoderinae	*Iridomyrmex dromus* Clark, 1938	iri.dro	38	253
Dolichoderinae	*Iridomyrmex mjobergi* Forel, 1915	iri.mjo	135	179
Dolichoderinae	*Iridomyrmex suchieri* Forel, 1907	iri.suc	450	403
Formicinae	*Melophorus* sp. JDM28	mel.j28	13	35
Formicinae	*Melophorus* sp. JDM472	mel.472	43	183
Formicinae	*Melophorus* sp. JDM791	mel.791	64	156
Formicinae	*Melophorus ludius* Forel, 1902	mel.lud	0	5
Formicinae	*Melophorus marius* Forel, 1910	mel.mar	9	7
Formicinae	*Melophorus turneri* Forel, 1910	mel.tur	425	749
Formicinae	*Melophorus turneri perthensis*	mel.tpe	36	2
Formicinae	*Melophorus wheeleri* Forel, 1910	mel.whe	27	87
Myrmicinae	*Monomorium laeve* Mayr, 1876	mon.lae	4	0
Myrmicinae	*Monomorium rothsteini* Forel, 1902	mon.rot	0	6
Myrmicinae	*Monomorium sordidum* Forel, 1902	mon.sor	281	240
Myrmicinae	*Pheidole hartmeyeri* Forel, 1907	phe.har	162	279
Myrmicinae	*Pheidole* sp. JDM177	phe.177	0	21
Ectatomminae	*Rhytidoponera metallica* Smith, F., 1858	rhy.met	50	267
Ectatomminae	*Rhytidoponera violacea* Forel, 1907	rhy.vio	1	3
Formicinae	*Stigmacros clivispina* Forel, 1902	sti.gla	0	3
Dolichoderinae	*Tapinoma* sp. JDM78	tap.j78	4	6
Dolichoderinae	*Tapinoma* sp. JDM981	tap.981	0	8
Myrmicinae	*Tetramorium* sp. 'near' *impressum*	tet.nri	3	11
Myrmicinae	*Tetramorium striolatum* Viehmeyer, 1914	tet.str	12	40

Supplementary Table 3. Functional traits used in the design of this study. For each of the functional traits recorded for ant species in this study, we provide a description and literature sources suggesting their relevance as indicators of habitat and resource use.

Trait	Description	Typical relation to resource use and environment preference
Weber's length	Posterior border of the metapleural lobe to the anterior border of the pronotum, exclusive of the anterior lip of the pronotum if present	Relates to habitat use and foraging mode sensu ref. [1], thermal and humidity tolerance [2,3].
Relative leg length	Sum of tibia length and femur length, excluding tarsi, relative to Weber's length	As per Weber's length. Additionally, may influence ant carrying capacity [3].
Relative head length	Head length measured from anterior clypeal margin to occipital margin, relative to Weber's length	Food item selection and processing. Relates to cranial musculature [4] and fluid intake rate [5].
Relative eye area	Area of eye, assuming ellipse (π × eye.length × eye.width), relative to head length	Broadly relates to resource niche [6], habitat preference and temporal partitioning [7].
Relative clypeus length	Measured along centre line of head in face view, from anterior to proximal margin including any anterior protusion (relative to head length)	Liquid food processing ability [8] and general resource choice.
Relative mandible length	From mandibular insertion to tip of apical tooth, relative to head length	Food item selection and processing [4,9].
Relative mandible width	Measured at widest point of mandible at 90 degrees from the margin, relative to head length	Food item selection and processing [4,9].
Relative scape length	From base of antennal insertion to the topmost margin of scape, relative to Weber's length	Likely size-grain and habitat use influences.
Polymorphism	Categorical factor; monomorphic, dimorphic, or polymorphic	General life history trait, potentially relating to food processing [sensu 9].
Cuticle tone	Measured under identical lighting conditions as the average greyscale value taken from 3 standard locations on the frons and 3 standard locations on the pronotum, of each individual. Values expressed as scale from 0 (black) to 100 (white), converted from a 0-256 greyscale pixel value.	May relate to thermal tolerance and partitioning across a thermal activity niche. [10,11].

Supplementary Table 4. Incidence of species in pitfall trap samples across each experimental block in 2012 prior to ant suppression treatments being imposed. Data support the analysis of species dominance and trait group allocation for the species represented in Extended Data Figure 1.

**Subfamily**	**Species**	**Species code**	**Trait group**	**Incidence in block 1**	**Incidence in block 2**	**Incidence in block 3**
Ponerinae	*Brachyponera lutea* Mayr, 1862	bra_lute	1	0.000	0.000	0.015
Myrmicinae	*Cardiocondyla atalanta* Forel, 1915	car.atlant	2	0.069	0.121	0.037
Dorylinae	*Lioponera clarki* Crawley, 1922	cer_clarki	2	0.000	0.008	0.000
Dorylinae	*Lioponera incontentus* Brown, 1975	cer.incon	1	0.008	0.008	0.022
Ponerinae	*Hypoponera congrua* Wheeler, 1934	hyp.congru	1	0.000	0.053	0.000
Dolichoderinae	*Iridomyrmex bicknelli* Emery, 1898	iri.bickne	3	0.221	0.106	0.133
Dolichoderinae	*Iridomyrmex brunneus* Forel, 1902	iri.brune	3	0.298	0.265	0.215
Dolichoderinae	*Iridomyrmex dromus* Clark, 1938	iri.dromus	3	0.168	0.114	0.030
Dolichoderinae	*Iridomyrmex mjobergi* Forel, 1915	iri.mjober	3	0.733	0.652	0.748
Dolichoderinae	*Iridomyrmex purpureus* Smith, 1858	iri_purpur	3	0.748	0.652	0.807
Dolichoderinae	*Iridomyrmex suchieri* Forel, 1907	iri.suchie	3	0.389	0.144	0.200
Formicinae	*Melophorus* sp. JDM28	mel.jdm28	4	0.176	0.136	0.037
Formicinae	*Melophorus* sp. JDM472	mel.jdm472	4	0.015	0.068	0.022
Formicinae	*Melophorus* sp. JDM791	mel.jdm791	4	0.053	0.000	0.059
Formicinae	*Melophorus ludius* Forel, 1902	mel.ludius	4	0.008	0.000	0.007
Formicinae	*Melophorus marius* Forel, 1910	mel.marius	4	0.038	0.008	0.030
Formicinae	*Melophorus turneri* Forel, 1910	mel.turner	4	0.916	0.856	0.867
Formicinae	*Melophorus turneri perthensis*	mel.turnpe	4	0.046	0.038	0.030
Formicinae	*Melophorus wheeleri* Forel, 1910	mel.wheele	4	0.595	0.303	0.378
Myrmicinae	*Chelaner anthracinus* Heterick, 2001	mon_anthra	3	0.000	0.008	0.007
Myrmicinae	*Monomorium laeve* Mayr, 1876	mon.laeve	2	0.008	0.023	0.015
Myrmicinae	*Monomorium rothsteini* Forel, 1902	mon.rothst	2	0.145	0.227	0.148
Myrmicinae	*Monomorium sordidum* Forel, 1902	mon.sordid	2	0.710	0.742	0.778
Myrmicinae	*Monomorium sydneyense* Forel, 1902	mon.sydj101	3	0.000	0.000	0.007
Dolichoderinae	*Ochetellus glaber* Mayr, 1862	och.gla019	3	0.008	0.000	0.000
Myrmicinae	*Pheidole ampla perthensis*	phe.amppe	5	0.954	0.939	0.970
Myrmicinae	*Pheidole hartmeyeri* Forel, 1907	phe.hartma	5	0.687	0.576	0.252
Ectatomminae	*Rhytidoponera metallica* Smith, F., 1858	rhy.metall	3	0.672	0.598	0.622
Ectatomminae	*Rhytidoponera violacea* Forel, 1907	rhy.violac	3	0.015	0.182	0.044
Myrmicinae	*Solenopsis clarki* Crawley, 1922	sol.clarki	1	0.023	0.023	0.022
Myrmicinae	*Strumigenys quinquedentata* Crawley, 1923	str.quiqu	1	0.008	0.000	0.015
Myrmicinae	*Tetramorium impressum,* Viehmeyer, 1925	tet.impres	3	0.206	0.114	0.126
Myrmicinae	*Tetramorium* sp. 'near' *impressum*	tet.nr_imp	3	0.015	0.015	0.007
Myrmicinae	*Tetramorium striolatum* Viehmeyer, 1914	tet.striol	3	0.084	0.083	0.170

Supplementary notes

Supplementary Note 1. Use of 84% confidence intervals in Figure 2

We adopted the approach of MacGregor-Fors & Payton [12] in using 84% confidence intervals as a visual aid in Figure 2, rather than a more commonly adopted 95% CI. These 84% CI allow a rapid visual comparison between mean rates of ecosystem processes in control and suppression plots, and are not used as a critical threshold in our statistical tests. Two non-overlapping 84% CI are a 'visual' indication of means that are significantly different at the $p = 0.05$ level, whereas two means with non-overlapping 95% CI differ significantly at the $p = 0.01$ level [12].

Supplementary Note 2. Species-level responses

Looking at species level responses in more detail, we find that some of the changes in performance rates of single functions could perhaps be attributed to species identity effects. Large *Iridomyrmex* species such as *I. purpureus*, known colloquially as meat ants, are often regarded as being the most competitively dominant members of Australian ant communities [e.g. 13], and were the most commonly observed species visiting our experimental nectaries and attacking surrogate herbivores in our plant protection assessment (*pers. obs*.). Other researchers looking to test competitive structuring in ant communities have experimentally removed meat ants and found that the functions they perform, or dominance in foraging rates, are generally taken up by subdominant, often functionally similar species [13,14]. To some degree this is also what we saw in our species functional importance analyses, with generally stronger responses from functionally similar, but subdominant species to those suppressed. When considered as a single function, rates of plant protection appear susceptible to the loss

of meat ants, suggesting that the local ant community has little redundancy in its ability to perform this function. Interestingly, two of the five strongest responding species for granivory were *Iridomyrmex* species, while the other three were *Melophorus* species. Although some *Melophorus* species are granivorous [15], *Iridomyrmex* species are not known to be granivorous ants. The observed responses, then, may be indicative of shifts in the competitive hierarchies within ant communities, whereby removal of meat ants releases other *Iridomyrmex* and *Melophorus* species from competition. Another good example of this type of indirect effect of suppression is where *Melophorus* species are increasing their functional performance rates in response to treatment. *Melophorus* are thermophyllic species that are able to forage at very high soil surface temperatures and may stratify their foraging activity thermally so as to avoid direct competition with highly dominant dolichoderines such as *I. purpureus* [13]. Where *I. purpureus* was suppressed in our treatment zones we found more *Melophorus* species than in control zones, foraging at lower temperatures (in early morning observations), and interacting with our seed baits during functional performance assays (*pers. obs.*). Gibb [16] found no cascading effects due to the suppression of meat ants, but the indirect effects of a competitive dominant on species performing other functions is similar to what is reported in the invasive species literature [e.g. 17], and likely to be expected in the context of a general ubiquity of competitive structuring in ant communities [18,19].

Supplementary Note 3. Methodological trial to assess feasibility of ant diversity manipulation

We conducted a small-scale methodological trial to assess the feasibility of removing the dominant ant species (with the highest incidence) in each of five functional groups defined in our functional trait analyses (i.e., *Iridomyrmex purpureus, Pheidole ampla perthensis, Tetramorium impressum, Monomorium sordidum,* and *Melophorus turneri*). We mapped

colonies of the five selected species using a combination of hand searching and following foraging ants after baiting with light-coloured cookie crumbs. *Monomorium sordidum* colonies proved difficult to find and led to concern that we could not reliably establish a sufficient level of suppression, so this species was discounted as a target for experimental manipulation. No remaining species in the *M. sordidum* functional group were of a high enough site-wide incidence (Table S2) to guarantee homogenous presence in both control and treatments zones. The thermophilic *Melophorus* species were all in the same functional group, and none was sufficiently active to map and remove in August and September. Colonies of *I. purpureus*, *P. ampla perthensis* and *T. impressum* all proved sufficiently easy to detect, either due to a propensity to nest in open ground or the characteristics of their nest spoil heap, and were therefore selected as the three target species for experimental suppression. The suppression of three species rather than five resulted in smaller reduction in overall trait dispersion, but still of a level that would allow comparisons of functional performance following manipulation of trait space. Suppression of the three species reduced trait dispersion by 12.48% (averaged across the three spatial blocks), while suppression of all five species would have reduced dispersion by a further 2.66%.

Supplementary Note 4. Functional assessments

Scavenging *(proportion of invertebrate carrion removed within 30 minutes)* – We assessed rates of ant scavenging for invertebrate carrion using timed removal rates of dead mealworms. We used freshly killed mealworms of between 8 and 12 mm in length. One mealworm was placed at each bait station, checked for ant activity every 15 minutes, and assessed for removal at 30 minutes.

Myrmecochory *(proportion of elaiosome-bearing seed removed within 60 minutes)* – We used *Acacia acuminata* seeds to assess ant-mediated seed dispersal, as this plant species occurs naturally at our site and its seeds are commonly removed by various ant species (pers. obs.). *Acacia acuminata* seeds are between 4 and 5 mm in length and compressed laterally, with a 3 mm elaiosome. We obtained fresh seed in the week before functional assessments began. All seeds were gamma irradiated to prevent later germination (Steritech AU, 50 kGy), as our study was conducted within a plant diversity experiment with quarantine regulations. One seed was placed at each bait station after ensuring it had an intact elaiosome, then checked for ant activity every 15 minutes, and assessed for removal at 60 minutes.

Granivory *(proportion of weed seed removed within 60 minutes)* – We assessed seed predation rates using a combination of two weed species common across the site, with ten *Lolium rigidum* and ten *Erodium* sp. seeds placed alternately along each transect. *Lolium rigidum* seeds are approximately 4 mm in length, and cylindrical with a 1.5 mm diameter. *Erodium* sp. seeds are the same diameter, but slightly shorter at 3.4 to 4 mm. Seeds were sourced on site in October 2014. One seed was placed at each bait station, checked for ant activity every 15 minutes, and assessed for removal at 60 minutes. Removal rates for the two weed seed species were pooled for the purposes of analysis.

Supplementary Note 5. Responses

Effective Number of Species calculation

Conventionally, most biodiversity-multifunctionality studies compare measures of function and multifunctionality against the degree of change in absolute species richness, as it is a simple robust measure that is translatable across studies. However, species richness ignores important variation in species commonness and rarity, which are also potentially important

predictors of functioning [e.g. 20], particularly in our context where we are likely to have treatment-driven changes in species dominance. Therefore, in addition to species richness, we use the effective number of equally-abundant species as a method of discriminating whether observed biodiversity-multifunctionality relationships still hold after accounting for effects of species relative abundance on functional rates. The effective number of equally-abundant species metric represents the hypothetical number of equally abundant species present in a community, and is calculated as:

$$\text{Effective Number of Species} = 1/\sum_{i=1}^{S} p_i^2$$

where S is species richness and p_i is the proportion of the community represented by species i [21,22]. In Hill series notation, effective number of equally-abundant species is the Hill-Simpson diversity index [23].

Raup-Crick dissimilarity calculation

The Chase et al. (2011) variant of the Raup-Crick dissimilarity metric is well suited for calculating dissimilarities without a ‘target’ reference community or single point of origin for distance calculations. The method compares species composition against a null model built from the entire meta-community, returning a value between -1 and +1 indicating whether an assemblage is compositionally more (positive values) or less (negative values) unique than would be expected by chance alone.

Supplementary Note 6. Analysis of SR-FR relationships

Generalized Additive Mixed Models (GAMMs)

Generalized Additive Mixed Models (GAMMs) with a Gaussian error distribution (and identity link function) were fitted using the ‘gamm’ function in package ‘mgcv’ [24]. A fixed

categorical effect was specified for treatment (control versus suppression) and a smooth function specified for species richness (SR). The continuous predictor SR was centred and scaled by 2 SD prior to analysis, as recommended by Gelman [25], using the 'rescale' function in the 'arm' package in R [26]. The GAMM basis function for the SR smooth was specified as a 'factor smooth' (bs = "fs") to allow separate smooth functions to be fitted for each level of the treatment factor. The GAMM settings used the default 'k=10' basis functions, smoothing penalization set at 'gamma=1.4' to avoid model over-fitting, and null-space shrinkage set at 'select=TRUE' to allow non-significant smooth functions to be removed during model fitting, as recommended by [24]. Random intercepts were specified in the model for 'block', to account for the split plot design of treatments paired within blocks, as well as for 'plot nested within block', to account for non-independence of multiple samples per plot.

We used a model comparison approach to determine the inclusion and significance of fixed effects in the best-fit GAMM, comparing five candidate models of increasing complexity: the null intercept-only model, a treatment-only model, a SR-only model, a model with different treatment-level intercepts but the same SR relationships, and a model with different treatment-level intercepts and different SR relationships for control versus suppression treatments. Candidate models were compared using the Akaike Information Criterion (AIC) in package 'AICcmodavg'[27].

Supplementary Table 5. Statistical output from path models built using generalized multilevel confirmatory path analyses in the 'piecewiseSEM' package in R. Separate path models were fitted for each functional response, as depicted in Figure 3. DF = degrees of freedom. The critical value represents the *t*- or *z*-value for the two-tailed test that the unstandardized partial regression estimate is significantly different from zero.

Response	Predictor	Unstandardised estimate	95 % Confidence interval	DF	Critical Value	P Value	Standardised Estimate
Species richness	Suppression treatment	0.272	0.065 – 0.479	8.00	6.666	0.033	0.273*

Granivory	Species richness	-0.705	-1.046 – -0.364	89.00	14.632	<0.001	-0.537***
Granivory	Suppression treatment	0.423	0.175 – 0.671	8.70	11.074	0.009	0.324**
Granivory	Richness × suppression	0.615	0.115 – 1.115	88.57	5.332	0.023	0.313*
Myrmecochory	Species richness	2.222	1.378 – 3.066	89.00	23.647	<0.001	0.585***
Myrmecochory	Suppression treatment	-0.054	-0.669 – 0.561	8.70	0.029	0.869	-0.014
Myrmecochory	Richness × suppression	0.482	-0.757 – 1.721	88.57	0.534	0.467	0.085
Plant protection	Species richness	0.857	0.462 – 1.252	90.35	16.989	<0.001	0.492***
Plant protection	Suppression treatment	-0.999	-1.321 – -0.677	8.58	36.772	<0.001	-0.577***
Plant protection	Richness × suppression	-0.153	-0.732 – 0.426	90.30	0.253	0.616	-0.059
Scavenging	Species richness	0.399	-0.128 – 0.926	89.52	1.981	0.163	0.216
Scavenging	Suppression treatment	0.060	-0.347 – 0.413	8.63	0.083	0.780	0.033
Scavenging	Richness × suppression	-0.201	-0.977 – 0.575	89.94	0.238	0.627	-0.073
Multifunctionality	Species richness	0.505	0.265 – 0.745	90.74	15.306	<0.001	0.504***
Multifunctionality	Suppression treatment	-0.180	-0.354 – 0.005	8.70	4.073	0.075	-0.181
Multifunctionality	Richness × suppression	0.170	-0.181 – 0.519	88.33	0.825	0.366	0.114

Supplementary references

1. Kaspari, M. & Weiser, M. D. The size–grain hypothesis and interspecific scaling in ants. *Funct Ecol* 13, 530–538 (1999).

2. Kaspari, M. Body size and microclimate use in Neotropical granivorous ants. *Oecologia* 96, 500–507 (1993).

3. Clémencet, J., Cournault, L., Odent, a. & Doums, C. Worker thermal tolerance in the thermophilic ant Cataglyphis cursor (Hymenoptera, Formicidae). *Insectes Soc* 57, 11–15 (2010).

4. Paul, J. Mandible movements in ants. *Comp Biochem Physiol A Mol Integr Physiol* 131, 7–20 (2001).

5. Paul, J. & Roces, F. Fluid intake rates in ants correlate with their feeding habits. *J Insect Physiol* 49, 347–357 (2003).

6. Weiser, M. D. & Kaspari, M. Ecological morphospace of New World ants. *Ecol Entomol* 31, 131–142 (2006).

7. Greiner, B. *et al.* Eye structure correlates with distinct foraging-bout timing in primitive ants. *Current Biology* 17, R879–R880 (2007).

8. Paul, J., Roces, F. & Hölldobler, B. How do ants stick out their tongues? *J Morphol* 254, 39–52 (2002).

9. Davidson, D. W. Size Variability in the Worker Caste of a Social Insect (Veromessor pergandei Mayr) as a Function of the Competitive Environment. *Am Nat* 112, 523 (1978).

10. Clusella Trullas, S., van Wyk, J. H. & Spotila, J. R. Thermal melanism in ectotherms. *J Therm Biol* 32, 235–245 (2007).

11. Frenette-Dussault, C., Shipley, B., Meziane, D. & Hingrat, Y. Trait-based climate change predictions of plant community structure in arid steppes. *Journal of Ecology* 101, 484–492 (2013).

12. MacGregor-Fors, I. & Payton, M. E. Contrasting Diversity Values: Statistical Inferences Based on Overlapping Confidence Intervals. *PLoS One* 8, (2013).

13. Andersen, A. N. & Patel, A. D. Meat ants as dominant members of Australian ant communities: an experimental test of their influence on the foraging success and forager abundance of other species. *Oecologia* 98, 15–24 (1994).

14. Gibb, H. & Hochuli, D. F. Removal experiment reveals limited effects of a behaviorally dominant species on ant assemblages. *Ecology* 85, 648–657 (2004).

15. Majer, J. D., Gove, A. D., Sochacki, S., Searle, P. & Portlock, C. A comparison of the autecology of two seed-taking ant genera, Rhytidoponera and Melophorus. *Insectes Soc* 58, 115–125 (2010).

16. Gibb, H. The effect of a dominant ant, Iridomyrmex purpureus, on resource use by ant assemblages depends on microhabitat and resource type. *Austral Ecol* 30, 856–867 (2005).

17. Rodriguez-Cabal, M. a, Stuble, K. L., Nuñez, M. a & Sanders, N. J. Quantitative analysis of the effects of the exotic Argentine ant on seed-dispersal mutualisms. *Biol Lett* 5, 499–502 (2009).

18. Hölldobler, B. & Wilson, E. O. *The Ants.* (Belknap Press Harvard, Cambridge, Massachusetts, USA., 1990).

19. Cerdá, X., Arnan, X. & Retana, J. Is competition a significant hallmark of ant (Hymenoptera: Formicidae) ecology? *Myrmecol News* 18, 131–147 (2013).

20. Soliveres, S. *et al.* Locally rare species influence grassland ecosystem multifunctionality. *Philosophical Transactions of the Royal Society B* (2016) doi:10.1098/rstb.2015.0269.

21. Jost, L. Entropy and diversity. *Oikos* 113, 363–375 (2006).

22. Chase, J. M. & Knight, T. M. Scale-dependent effect sizes of ecological drivers on biodiversity: Why standardised sampling is not enough. *Ecol Lett* 16, 17–26 (2013).

23. Roswell, M., Dushoff, J. & Winfree, R. A conceptual guide to measuring species diversity. *Oikos* 130, 321–338 (2021).

24. Wood, S. *Generalized Additive Models: An Introduction with R* . (Chapman and Hall, 2017).

25. Gelman, A. Scaling regression inputs by dividing by two standard deviations. *Stat Med* 27, 2865–2873 (2008).

26. Gelman, A. & Su, Y.-S. Data Analysis Using Regression and Multilevel/Hierarchical Models. R package version 1.10-1. https://CRAN.R-project.org/package=arm. Preprint at <https://CRAN.R-project.org/package=arm> (2024).

27. Mazerolle, M. J., Marc, M. & Mazerolle, J. Model Selection and Multimodel Inference Based on (Q)AIC(c) Depends R (>= 3.2.0). Preprint at (2023).
