# Supplementary material for: Functional redundancy compensates for decline of dominant ant species
Source: Nat Ecol Evol. 2025 Apr 22;9(5):779–88. doi: 10.1038/s41559-025-02690-y (PMC12066353; doi:10.1038/s41559-025-02690-y)
Supplement: Supplementary file 2 — Reporting Summary [file 41559_2025_2690_MOESM2_ESM.pdf]

nature portfolio

Corresponding author(s): Peter Yeeles
Raphael K Didham

Last updated by author(s): Mar 10, 2025

Reporting Summary

Nature Portfolio wishes to improve the reproducibility of the work that we publish. This form provides structure for consistency and transparency in reporting. For further information on Nature Portfolio policies, see our Editorial Policies and the Editorial Policy Checklist.

Statistics

For all statistical analyses, confirm that the following items are present in the figure legend, table legend, main text, or Methods section.

n/a	Confirmed	
☐	☒	The exact sample size (n) for each experimental group/condition, given as a discrete number and unit of measurement
☐	☒	A statement on whether measurements were taken from distinct samples or whether the same sample was measured repeatedly
☐	☒	The statistical test(s) used AND whether they are one- or two-sided<br>*Only common tests should be described solely by name; describe more complex techniques in the Methods section.*
☐	☒	A description of all covariates tested
☐	☒	A description of any assumptions or corrections, such as tests of normality and adjustment for multiple comparisons
☐	☒	A full description of the statistical parameters including central tendency (e.g. means) or other basic estimates (e.g. regression coefficient) AND variation (e.g. standard deviation) or associated estimates of uncertainty (e.g. confidence intervals)
☐	☒	For null hypothesis testing, the test statistic (e.g. F, t, r) with confidence intervals, effect sizes, degrees of freedom and P value noted<br>*Give P values as exact values whenever suitable.*
☒	☐	For Bayesian analysis, information on the choice of priors and Markov chain Monte Carlo settings
☐	☒	For hierarchical and complex designs, identification of the appropriate level for tests and full reporting of outcomes
☐	☒	Estimates of effect sizes (e.g. Cohen's d, Pearson's r), indicating how they were calculated

Our web collection on statistics for biologists contains articles on many of the points above.

Software and code

Policy information about availability of computer code

Data collection	No software was used for data collection.
Data analysis	All analyses were performed with the open source R (version 4.1.2).<br><br>We used the 'lme4' package to test for an effect of ant suppression treatment and the 'piecewiseSEM' package for piecewise structural equation models. We used the software 'Impact' (version 1.0) to compare species abundances with functional performance measures, and 'multifunc' for analysing functional performance data. Plots were generated using the ggplot2 R package.<br><br>Code is available to download from Figshare: https://doi.org/10.6084/m9.figshare.27998150 (this DOI is referenced in the manuscript).

For manuscripts utilizing custom algorithms or software that are central to the research but not yet described in published literature, software must be made available to editors and reviewers. We strongly encourage code deposition in a community repository (e.g. GitHub). See the Nature Portfolio guidelines for submitting code & software for further information.

Data

Policy information about availability of data

All manuscripts must include a data availability statement. This statement should provide the following information, where applicable:

- Accession codes, unique identifiers, or web links for publicly available datasets
- A description of any restrictions on data availability
- For clinical datasets or third party data, please ensure that the statement adheres to our policy

Raw data are available to download from Figshare: https://doi.org/10.6084/m9.figshare.27998150 (this DOI is referenced in the manuscript).

Field-specific reporting

Please select the one below that is the best fit for your research. If you are not sure, read the appropriate sections before making your selection.

☐ Life sciences ☐ Behavioural & social sciences ☒ Ecological, evolutionary & environmental sciences

For a reference copy of the document with all sections, see nature.com/documents/nr-reporting-summary-flat.pdf

Ecological, evolutionary & environmental sciences study design

All studies must disclose on these points even when the disclosure is negative.

Study description	This study uses an experimental manipulation of ant species diversity to investigate the relative importance of functional redundancy versus complementarity in conferring the stability of multi-functional ecosystem function performance. We suppressed the abundance of dominant ant species from multiple trait groupings and measured subsequent changes in the rates of 4 important ecosystem processes.
Research sample	Our research samples consisted of ant specimens and records of the ecological processes they performed.
Sampling strategy	We used 4 pitfall traps per plot to assess invertebrate community responses to the ant suppression treatment. Pitfall cups were 69mm diameter and 62mm in depth, filled with 50 ml of a 50:50 water and propylene glycol preservative. Traps were left open for one week durations.
Data collection	Observations were carried out only in calm and sunny weather, from 8:30 AM to 5:00 PM.
Timing and spatial scale	Sampling and observations took place every three months for one year from treatment imposition, in November 2014, and February, May and November 2015.
Data exclusions	No data were excluded from the analysis.
Reproducibility	All data necessary to repeat the analyses will be made publicly available.
Randomization	The site layout within the context of a broader ecosystem function experiment combined with method of treatment application did not allow randomization of site-treatment allocation.
Blinding	Information regarding treatment level was blinded during taxonomic identification of insect voucher specimens.

Did the study involve field work? ☒ Yes ☐ No

Field work, collection and transport

Field conditions	The main habitat type is restoring Eucaluptus forest. The climate is Mediterranean, with hot dry summers and cool wet winters. Median annual precipitation in this area is 443 mm, mainly concentrated in winter between April and September The average annual temperature ranges from 10.4 °C mean minimum to 23.5 mean maximum °C.
Location	The study was carried out in at the Ridgefield Multiple Ecosystem Services experiment, located near Pingelly in Western Australia, Australia.
Access & import/export	No permits were required to perform this work.
Disturbance	No disturbance was caused in the study sites.

Reporting for specific materials, systems and methods

We require information from authors about some types of materials, experimental systems and methods used in many studies. Here, indicate whether each material, system or method listed is relevant to your study. If you are not sure if a list item applies to your research, read the appropriate section before selecting a response.

Materials & experimental systems

n/a	Involved in the study	
☒	☐	Antibodies
☒	☐	Eukaryotic cell lines
☒	☐	Palaeontology and archaeology
☐	☒	Animals and other organisms
☒	☐	Human research participants
☒	☐	Clinical data
☒	☐	Dual use research of concern

Methods

n/a	Involved in the study	
☒	☐	ChIP-seq
☒	☐	Flow cytometry
☒	☐	MRI-based neuroimaging

Animals and other organisms

Policy information about studies involving animals; ARRIVE guidelines recommended for reporting animal research

Laboratory animals	*For laboratory animals, report species, strain, sex and age OR state that the study did not involve laboratory animals.*
Wild animals	This study involved sampling wild ants. Ants were collected using pitfall traps filled with liquid preservative, which killed ants upon capture. A list of species captured is included in Supplementary Table S2. Ants were preserved either dry, on card points, or wet in ethanol and identified in a laboratory after fieldwork. Curated specimens were lodged at the West Australian Museum.
Field-collected samples	*For laboratory work with field-collected samples, describe all relevant parameters such as housing, maintenance, temperature, photoperiod and end-of-experiment protocol OR state that the study did not involve samples collected from the field.*
Ethics oversight	As this project only involved the sampling of ants, there was no ethics oversight required under Australian law.

Note that full information on the approval of the study protocol must also be provided in the manuscript.
